# Natural killer cells- from innate cells to the discovery of adaptability

**DOI:** 10.3389/fimmu.2023.1172437

**Published:** 2023-05-18

**Authors:** Débora Basílio-Queirós, Eva Mischak-Weissinger

**Affiliations:** Hannover Medical School, Department of Hematology, Hemostasis, Oncology and Stem Cell Transplantation, Hannover, Germany

**Keywords:** natural killer cells, cytotoxic lymphocytes, adaptive natural killer cells, viral infections, immune, Cytomegalovirus

## Abstract

Natural Killer (NK) cells have come a long way since their first description in the 1970’s. The most recent reports of their adaptive-like behavior changed the way the immune system dichotomy is described. Adaptive NK cells present characteristics of both the innate and adaptive immune system. This NK cell subpopulation undergoes a clonal-like expansion in response to an antigen and secondary encounters with the same antigen result in an increased cytotoxic response. These characteristics can be of extreme importance in the clinical setting, especially as adoptive immunotherapies, since NK cells present several advantages compared other cell types. This review will focus on the discovery and the path to the current knowledge of the adaptive NK cell population.

## Background

The immune system is classically divided in two branches, the innate and adaptive immunity according to the cellular characteristics. The innate branch of the immune system offers a fast but unspecific response against pathogens. The adaptive immune system recognizes antigens/pathogens and cells are clonally expanded and, in the case of antigen-specific B-cells, even modified to obtain higher levels of antigen specificity (1–5).

Innate lymphoid cells (ILCs) comprise a variety of common lymphoid progenitor-derived cells that do not express somatically rearranged antigen-specific receptors and play important roles in immune homeostasis. One subset of ILCs are Natural Killer (NK) cells (6–8). In humans, they comprise between 5 to 20% of circulating lymphocytes (9). These immune cells have very diversified functions that can range from their classic innate anti-tumor and anti-viral functions, to regulatory roles involved in the modulation of other immune cells as well as tissue growth promotion (10).

NK cells were first described in the early 1970’s when a significant natural cytotoxicity to target cells was observed in lymphocytes from unimmunized mice and normal human lymphoid tissue (10–13). Until then, it was believed that non-T cell-mediated cytotoxicity was only achieved by antibody-dependent-cell-cytotoxicity (ADCC) or macrophages (14–16). R. Kiessling et al. were able to exclude a possible involvement of macrophages, T and B cells. This was achieved by the combined results observed in athymic nude mice and inability to identify the aforementioned cells. A new and unique cell population able to kill mouse tumor cells was hence recognized. Even though very little was known about this cell population, this observation opened the door to a brand-new world in immunology (11). We have come a long way in our knowledge about NK cells since these days.

In the following sections, this review aims to summarize the evolution in NK cells knowledge from their discovery to the detection of their adaptive phenotype.

## Natural killer cells biology and receptors

Natural Killer cells were the first member of the ILC family to be discovered. They possess a lymphoid developmental origin and a T-bet-regulated rapid cytokine production activation profile. However, this activation does not lead, generally, to immunological memory. Furthermore, NK cells lack clonally rearranging antigen receptors (17). This was demonstrated in mice, where RAG-1 or RAG-2 disrupted genes did not affect the development of functional NK cells (18, 19). It is known that NK cells are involved in the immune response to several pathogens and are also involved in the first line of defense against tumor cells without prior sensitization (20–23). NK cells were normally characterized according to their morphology, as large granular lymphocytes and phenotypically, defined by the surface expression of the cluster of differentiation (CD)56 and lack of CD3 (24). Another definition of mature conventional NK cells relies on exclusion criteria describing NK cells as non-T, non-B lymphoid cells with the ability to rapidly produce interferon (IFN) -gamma upon stimulation with pathogens (25).

NK cells are bone marrow derived and can be found in the peripheral circulation, but are also present in other lymphoid and non-lymphoid organs (26). Two major distinct populations of circulating NK cells are recognized according to the surface expression of CD56 and CD16. It is considered that CD56^dim^CD16^+^, corresponding to approximately 90% of peripheral blood NK cells, represent a more cytotoxic population while the CD56^bright^CD16^dim/-^, corresponding to the remaining 10%, have a more regulatory role, being highly involved in the production of cytokines (27–29). There is, however, a much higher count of NK cells subpopulations when it comes to the different receptors expressed by these cells. For instance, in 2009, Milush et al. reported new subpopulations of NK cells based on the expression of CD7, a marker shared with T and pre-B cells. The surface expression of this receptor was associated with the co-expression of other NK cell-associated receptors such as Killer-Immunoglobulin Receptors (KIRs) or Natural Cytotoxicity receptors (NCRs) (30). With the help of technological advancement, and the possibility to simultaneously analyze more than 30 parameters, Horowitz et al. indicated that at least 30 000 different phenotypes of conventional peripheral blood NK cells can be present at any given point (31). The great diversity of NK cells populations among each individual or patient, comprising different functions and degrees of maturation, may result in varied responses. The challenge to current research is to understand how these different populations can be exploited in the design of specific therapies.

Different NK cell subpopulations can have different, more or less specialized, functions and responses. Nonetheless, a very strict balance between activating and inhibitory receptors regulates all NK cells. Contrary to what happens with adaptive cells, NK cell receptors are germline-encoded with no requirements for recombination and the activation status of NK cells is determined by the balance between activating or inhibitory signals. A key regulator of NK cell activation is the constitutively expressed Major Histocompatibility Complex (MHC) class I. MHC class I molecules bind to the inhibitory receptors, including members of the Killer Immunoglobulin-like Receptor (KIR) family and prevent NK cell activation (32). However, infected, tumor-transformed or stressed cells, undergo a downregulation or even loss of MHC class I expression. This will result in a lack of inhibitory, or rather a prevalence of activating signals, tipping the balance towards the activation of NK cells (33, 34). This mechanism allows for the preservation of ‘self’ while engaging in the elimination of the ‘missing-self’ (32).

The activating receptors of NK cells include receptors belonging to the C-type lectins family and NCRs mentioned above. NCRs (e.g. NKp30, NKp44, NKp46 and NKp80) arm NK cells with the ability to effectively kill tumor-transformed cells (35–38). The key role of NCRs in the elimination of tumor cells is well established and can be demonstrated, for instance, by an ineffective clearance of certain tumors in the absence of NKp46 in *in vitro* and *in vivo* models (39–41). Furthermore, NCRs are also implicated in the control and clearance of pathogens. For instance, NKp46 is essential to the elimination of virus and bacteria *in vivo*. This was demonstrated by the inability of NKp46-deficient mice to recognize and eliminate influenza-infected cells expressing NKp46-ligands or as observed by the reduced activation and IFN-gamma production during the early stages of *Streptococcus pneumoniae* infections (42, 43). In regards to C-type lectins, CD94 forms covalent bonds with members of the NKG2 family (A, C, D and E) and forms heterodimers expressed by NK cells and a subset of cytotoxic T lymphocytes (44).

An overview of some inhibitory and activating receptors expressed at the surface of NK cells is summarized in **Table 1**.

**Table 1:** Human NK cell Activating and Inhibitory Receptor Families.

Family	Members	Molecular Structure	Function
**KIR**	KIR2DL1 (45)	Immunoglobulin Superfamily	Inhibitory
	KIR2DL2 (46)		Inhibitory
	KIR3DL1 (47)		Inhibitory
	KIR3DL3 (48)		Inhibitory
	KIR2DS1 (49)		Stimulatory
	KIR2DS2 (50)		Stimulatory
	KIR2DS4 (51)		Stimulatory
**NCR**	NKp30 (35)	Immunoglobulin Superfamily	Stimulatory
	NKp46 (37)		Stimulatory
	NKp80 (52)		Co-stimulatory
	NKp44 (38)		Stimulatory
**NKG2 family**	CD94/NKG2A (53)	C-type Lectins	Inhibitory
	CD94/NKG2C (54)		Stimulatory
	CD94/NKG2D (55)		Stimulatory
	CD94/NKG2E (56)		Stimulatory

Besides their ability to recognize and kill tumor and virus-infected cells, NK cells are also able to interact with other cell types and orchestrate the adaptive immune response. Most notably, NK cells are able to modulate Dendritic cells (DCs), macrophages and T cell (57–59). One particular example of this modulatory capability is seen, for instance, by the ability of NK cells to edit the maturation of autologous DCs through NKp30-mediated elimination of aberrant or immature DCs while sparing fully matured DCs. This results in a correct DC-priming and subsequent antigen-specific T cell response (60, 61).

## Natural killer cells; innate or adaptive?

By definition, memory or adaptive cells are a population of long-lived, self-renewing immune cells with the ability of antigen-specific recognition and memory formation. For many years, conventional NK cells were described as short-lived innate lymphocytes lacking antigen specificity. However, in recent years this idea has been challenged. Murine studies showed the ability of NK cells to acquire selective memory. This phenomenon was demonstrated by hapten-induced contact hypersensitivity and recall responses in challenged mice, lacking mature T and B cells. This feature was previously widely accepted as a T cell effect. Furthermore, this NK cell response persisted for several weeks and was able to discriminate between different haptens. This assembles three hallmarks characteristics of adaptive immunity: i) acquired activity, ii) antigen specificity and iii) long-lived memory cells (62). The hapten-specific response was further characterized by Paust et al., while the NK cell memory development in response to different viruses and possible influence of the host’s genetic background was also assessed. To elucidate this memory function, non-infectious virus-like particles containing proteins from influenza or HIV and UV-inactivated vesicular stomatitis virus were used. Immunization of naïve Rag1^−/−^ mice and subsequent adoptive transfer of purified NK cells into naïve Rag2^−/−^Il2rg^−/−^ mice resulted in a vigorous and sustained response at challenge 4 weeks later. This response was virus-specific and restricted to hepatic NK cells isolated from immunized mice, while splenic NK cells were unresponsive. Furthermore, this NK cell-specificity persisted for at least 4 months (63). Another study described the ability of NK cells to develop a memory-like behavior following cytokine stimulation. The *in vitro* stimulation consisted of a combination of interleukin (IL)-12, IL-15 and IL-18. NK cells were then adoptively transferred to naïve Rag1^−/−^ mice and re-stimulation with IL-12 and IL-15 or *via* the engagement of activating NK cell receptors with antibodies led to a robust IFN-gamma secretion. Furthermore, the often termed ‘memory-like’ NK cells in this experimental setting, produced significantly higher levels of IFN-gamma which was detected for at least 3 weeks post-adoptive transfer of pre-activated NK cells (64).

## The particular role of Cytomegalovirus

Gumá et al. described the expansion of a population of NK cells in response to cytomegalovirus (CMV)-infected fibroblasts in 2006 and this set off increasing interest in NK cell function thus accumulating knowledge over the last decade (21). The NK relationship with CMV has for long been appreciated by the observation of the high susceptibility to CMV-infections in both humans and mice lacking functional NK cells (65–69).

Murine models facilitated the intensive study of the NK cell-CMV molecular mechanisms. This was largely simplified by the identification of both receptor and ligand involved in the NK cell memory formation. The murine NK cell activating receptor Ly49H recognizes the m157 protein expressed on infected cells (70). Multiple factors have been identified as essential for the NK cell memory development during murine CMV (MCMV) infection at different stages. These include IL-12/STAT4 signaling, IL-18, miR-155, the Bim protein, the Zbtb32 transcription factor, recombination-activation genes and mitophagy (71–76). In MCMV, contrary to Paust et al. hapten-induced contact hypersensitivity study, adaptive NK cells were found in the liver but also in the spleen, lung, kidney, blood circulation and other lymphoid tissues. This population of NK cells underwent an expansion phase, followed by a contraction phase after resolution of viral infection and ultimately resulted in the generation of long-lived “memory” NK cells that were more protective during a second encounter with this pathogen (77, 78).

In humans, it is now known that this particular NK cell behavior is associated to the non-classical MHC class I molecule HLA-E and its antigenic presentation of human CMV (HCMV) viral peptide(s) to NKG2C, which is a C-type lectin that covalently bonds to CD94 (79). In HCMV, conflicting information regarding the expansion of this cell population was reported. Several studies described the expansion of NKG2C^+^ NK cells in other viral infections. These ranged from HIV, Hantavirus, Chikungunya virus, Hepatitis B virus and Epstein-Barr virus (80–86). Interestingly however, the expansion of NKG2C^+^ NK cells was almost completely limited to the HCMV seropositive population in all the studies in which CMV status was assessed. This suggests that HCMV is the common denominator in the expansion of NKG2C^+^ NK cells (80, 81, 85, 86). This supported the previous observation of Gumá et al. that HCMV influences the shaping of the NK cell receptor repertoire. Furthermore, the expansion of NKG2C^+^ NK cells was not seen in the context of other herpesviruses infections. Thus, HCMV may be unique in its ability to recall NKG2C^+^ NK cells responses (66, 87).

The diversity within NK cells may be the result of what has been described as an ‘arms race’ between NK cells and viruses (88) [74]. CMV infection results in the modulation and downregulation of MHC class I molecules on the surface of infected cells in an attempt to escape recognition by T cells (89). In fact, in the case of infected-cells being eliminated by class I-restricted CD8^+^ cytotoxic T lymphocytes (CTLs), pathogens that attenuate class I expression will become invisible, at least temporarily, to CTLs and therefore, have a selective advantage. In CMV-infection, this is achieved by different approaches. For example, the transport of peptides produced in the cytosol can be affected by the HCMV US6 gene attacking the TAP complex and preventing class I heterodimers from binding (90). Other strategies may be related to the retention or destruction of class I molecules. The US3 gene product binds to class I molecules sequestering them in the endoplasmic reticulum (91). Furthermore, US2 and US11 products bind to class I molecules and redirect the class I heavy chain to the cytosol, reversing the process by which the chain is inserted in the endoplasmic reticulum (92). This CTL evasion mechanism can, however, lead to the engagement of a NK cell response. NK cells are usually prevented from activation by the engagement of inhibitory receptors by self-MHC products. Certain viruses are capable of successfully reducing the surface expression of MHC class I, while controlling NK cell activation by the expression of class I homologues that will serve as decoys for NK cells. In the case of CMV, examples of this are UL18 in humans and m144 in mouse. In the particular case of UL18, this viral homologue binds to inhibitory receptors with higher affinity than MHC class I (93–95). Furthermore, the HCMV UL40 open reading frame, contains a segment that is homologous to the HLA-E binding leader peptide (96). However, the efforts of CMV to circumvent the immune system go one step even further. HCMV harbors a unique IL-10 homolog (cmvIL-10) which is able to compete with human IL-10 for binding sites (97). This viral cmvIL10 may facilitate HCMV replication by suppressing or tampering with inflammatory responses. Furthermore, a study by Chang et al. showed that the production of cmvIL-10 inhibits the production of IL-12 and tumor necrosis factor (TNF)-alpha by DCs in a concentration-dependent manner (98). As IL-12 promotes the cytotoxic and proliferative capacity of NK cells, cmvIL-10 may inhibit or delay the activation NK cells both directly and indirectly. All these mechanisms employed by CMV to evade NK cell-recognition point to the importance of NK cells in the control of CMV.

Interestingly however, cmvIL-10 was shown to induce NK cell activation and the increased NK cell cytotoxicity was triggered by several activating receptors. cmvIL-10 binds to the IL-10 receptor (IL-10R). Even though IL10-R is expressed at low levels on NK cells, the production of a virokine able to activate NK cells by HCMV is puzzling (99). One may hypothesize that the low level of activation promotes a pro-inflammatory environment beneficial for HCMV infection and/or dissemination all the while ensuring that a strong enough trigger does not result in an effective antiviral response.

## Epigenetic profile towards adaptivity

The NK cell population is one of great heterogeneity both in phenotype and function. CMV was shown to directly influence the NK cell population and lead to lasting alterations (87, 100). Furthermore, CMV was also shown to influence the outcome after hematopoietic stem cell transplantation of acute and chronic myeloid leukemia patients since CMV reactivation controlled by CMV-CTLs led to decreased relapses in these patients (101, 102). A recent study by our group demonstrated that adaptive NK cells have the ability to recruit T cells when cultured with CMV-infected target cells (103). However, at the molecular level, the receptor modulation and NK cell differentiation has not yet been described. A study by Schlums et al. shed some light on these questions. The analysis of 196 healthy adults showed that the CD56^dim^ NK cell population from many donors lacked expression of the adaptor protein FcεRɣ, the tyrosine kinase SYK and the intracellular adaptor protein EAT-2 (50.4% of HCMV seropositive donors vs 10.1% of HCVM seronegative donors). They demonstrated that this effect correlated to HCVM seropositivity regardless of sex and age of the donor, B cell or myeloid-cell-related signaling proteins. Furthermore, this was observed only in acute HCMV infection (*de novo* or reactivation). The lack of FcεRɣ, SYK and EAT-2 correlated with phenotypic characteristics of adaptive NK cells, namely, the expression of NKG2C and absence or reduced expression of NKp30. Deficiency for EAT-2 and FcεRɣ was shown to be the result of hypermethylation in their promoter region (104). Similar results were observed for SYK by Lee et al. (105). Moreover, genome-wide analysis of DNA methylation comparing canonical and adaptive NK cells to T cells were performed. This revealed that the methylation profile of adaptive NK cells was closely related to the one of effector T cells. Adaptive NK cells had 2372 differentially methylated regions (DMRs) compared to canonical NK cells while only differing in 61 DMRs when compared to CD8^+^ T cells. Furthermore, this methylation profile was responsible for the regulation of the gene expression on adaptive NK cells (104).

## Origins of natural killer cell memory

**Figure 1:**
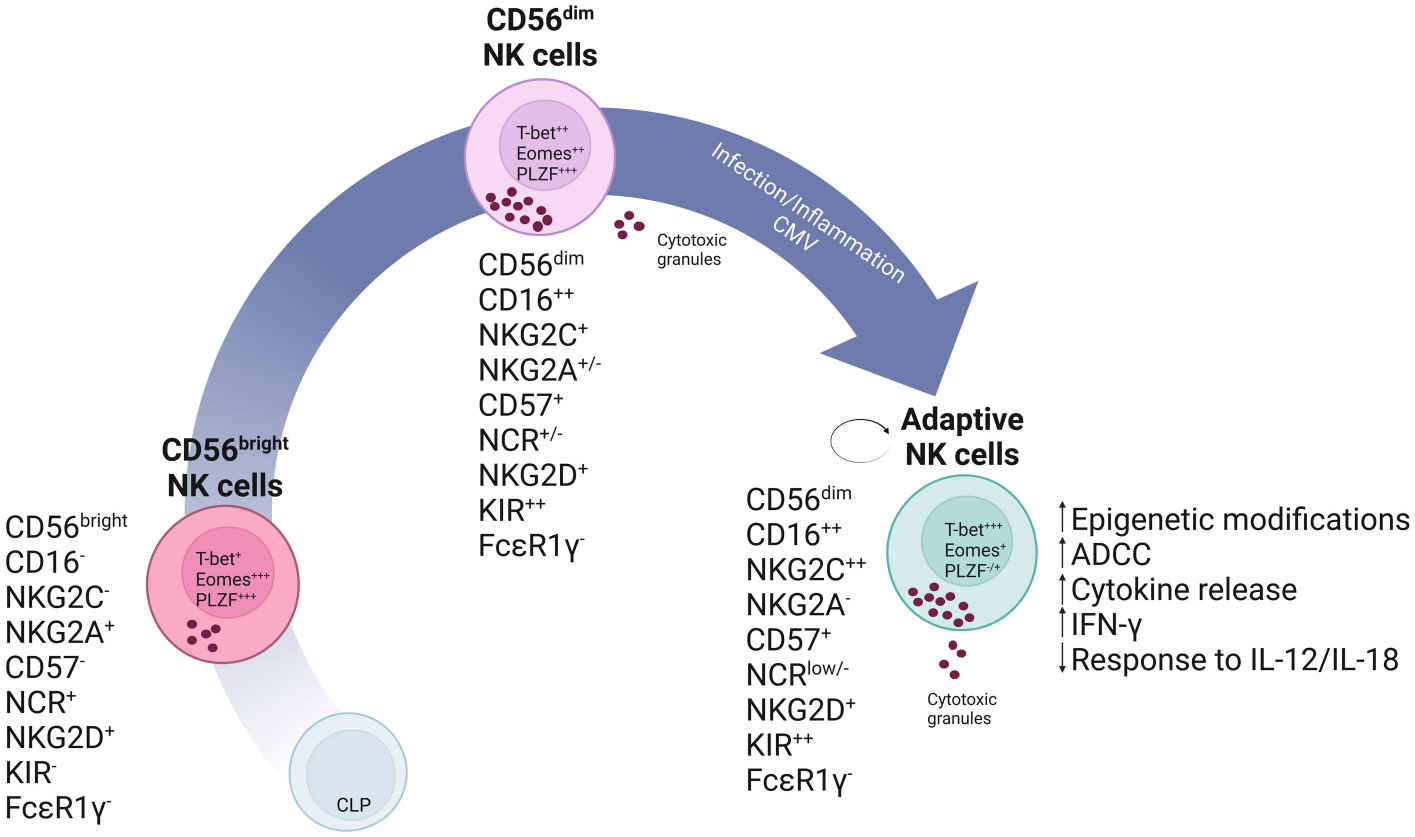
Schematic representation of a linear model of NK cell development. In this model it is theorized that, common lymphoid progenitor (CLP) cells commit into NK cell precursors and mature into CD56bright NK cells. CD56bright NK cells are less differentiated and express high levels of the inhibitory receptor NKG2A and natural cytotoxicity receptors (NCRs). These cells will give rise to mature CD56dim NK cells which have enhanced cytotoxic capabilities as seen, for instance, by increased production of cytotoxic granules and CD16 expression. These can subsequently further differentiate into self-renewing adaptive NK cells expressing high levels of NKG2C and Killer Immunoglobulin-like receptors (KIR) as the result of specific stimuli, which may include a combination of cytokines or CMV infection.

Nagler et al. originally described CD3^-^CD56^dim^ and CD3^-^CD56^bright^ as subpopulations of NK cells in different differentiation stages, where the CD3^-^CD56^bright^ mature overtime to the CD3^-^CD56^dim^ population (28). The GATA-binding factor 2 (GATA-2) transcription factor is necessary for hematopoietic stem and progenitor cell survival and proliferation. Its haploinsufficiency results in deficiencies in Dendritic, B and NK cells and leads to clinical symptoms of immunodeficiency, lymphedema and even myelodysplastic syndrome (106, 107). A more recent analysis and characterization of symptomatic GATA-2 (+/- or mutation) patients’ showed that persisting NK cells displayed characteristics and functional properties of adaptive NK cells. Taken this into account, adaptive NK cells would be able to persist in the absence of their CD56^dim^ precursors raising questions onto the ontogeny of adaptive NK cells. Characteristics of persisting NK cells included a decreased expression of the promyelocytic leukemia zinc finger (PLZF) and T-box transcription factors (T-bet), maintained Fc receptor expression and cytotoxic capacity in response to antibody-coated target cells, as well as, degranulation capacity and IFN-gamma and TNF secretion. Furthermore, the persistence of this cell population despite the GATA-2 insufficiency that results in the abolishment of canonical NK cells demonstrates the considerable increased longevity of adaptive NK cells (108). Another study provides further evidence into the longevity of adaptive NK cells and further supports a self-renewal hypothesis. Here, paroxysmal nocturnal hemoglobinuria patients have an X-linked acquired Phosphatidylinositol N-acetylglucosaminyltransferase subunit A (PIGA) mutation. PIGA encodes a protein required for the synthesis of glycosylphophatidylinositol (GPI) anchors. These loss-of-function mutations occur in hematopoietic stem and progenitor cells, and result in the production of hematopoietic cells deficient in GPI-anchored membrane proteins (109). Progression of the disease from a GPI^pos^/GPI^neg^ mixed chimerism towards a virtually exclusive GPI^neg^ lineage can give insight into the development of adaptive NK cells. Results from 15 patients suggested that CD56^dim^ NK cells may persist and propagate independently of CD56^bright^. Furthermore, the majority of adaptive NK cells where GPI^pos^, while canonical NK cells were vastly GPI^neg^. Consistent with prior studies, GPI^pos^ adaptive NK cells showed marked reduction in IFN-gamma production in response to co-stimulation with IL-12 and IL-18 innate cytokines while maintaining degranulation capacity upon engagement of the low affinity Fc receptor CD16. This indicates that the described long-lived GPI^pos^ NK cells are functionally comparable to adaptive NK cells (110). These studies suggest a peripheral pathway for the maintenance of CD56^dim^ adaptive NK cells independent of hematopoietic stem and progenitor cells production and also independent of CD56^bright^ precursor (108, 109). A linear model representation of the developmental pathway of (adaptive) NK cells is shown in **Figure 1**.

## Importance in health and disease

Natural Killer cells play a role in the most varied biological processes and are involved in both health and disease. These range from combating infections, cancer, autoimmune disorders and even in the maintenance of normal pregnancy. NK cells contribute to the control of viral infections by both, direct cytotoxicity against virally-infected cells, but also by the production of cytokines that may control viral replication and regulate an adaptive immune response (103). They are also involved in the elimination of other types of infection that include infections caused by intracellular bacteria, fungi and some protozoa (111). One other important function of Natural Killer cells is the elimination of tumor cells. In this scenario, NK cells’ most likely role is the surveillance and elimination of malignant cells in order to prevent the formation of tumors. In regards to autoimmune disorders such as lupus erythematosus, NK cells can play a role in tolerance induction, by decreased numbers and activity, increased proportion of CD56^bright^ cells and impaired cytotoxicity (112–114). NKp46, a NCR NK cell’s receptor, has been involved in the pathophysiology of type 1 diabetes while in rheumatoid arthritis, a subset of Natural Killer cells is greatly expanded in patients with inflamed joints (115, 116). NK cells also play an essential role in the efficacy of some vaccines and immunotherapies, such as seen as the result of, for instance, the BCG vaccine (117). NK cells further cooperate in vaccine efficacy through their interaction with vaccine-induced antibodies with NK cell-activation properties on top of the virus-neutralizing properties (118). All this demonstrates the immense possibilities of NK cells in clinical applications.

## Conclusion

If NK cells are believed to be an evolutionary bridge between innate and adaptive immunity, it is not surprising that they will exhibit features of both. Implications of NK cells in such mixed processes and the recent discovery of their adaptive profile demonstrates that the exploitation of this cell population and their use in possible therapeutic approaches can have great implications in health and disease. For these reasons, further research in the full range of possible applications of adaptive NK cells is warranted.

## Author contributions

DB-Q contributed to the concept and design of the article. EM-W revised the article critically for important intellectual content. All authors contributed to the article and approved the submitted version.

## References

[1] Chaplin DD. Overview of the immune response. J Allergy Clin Immunol (2010) 125(2 Suppl 2):S3–23. doi: 10.1016/j.jaci.2009.12.980 20176265 PMC2923430

[2] Charles A Janeway J. How the immune system works to protect the hostfrom infection: a personal view. PNAS (2001) 18(13):7461–8. doi: 10.1073/pnas.131202998 PMC34691 11390983

[3] Dranoff G. Cytokines in cancer pathogenesis and cancer therapy. Nat Rev Cancer (2004) 4(1):11–22. doi: 10.1038/nrc1252 14708024

[4] Lakkis FG Sayegh MH. Memory T cells: a hurdle to immunologic tolerance. J Am Soc Nephrol (2003) 14(9):2402–10. doi: 10.1097/01.ASN.0000085020.78117.70 12937320

[5] Nemazee D. Receptor selection in b and T lymphocytes. Annu Rev Immunol (2000) 18:19–51. doi: 10.1146/annurev.immunol.18.1.19 10837051 PMC3822044

[6] Artis D Spits H. The biology of innate lymphoid cells. Nature (2015) 517(7534):293–301. doi: 10.1038/nature14189 25592534

[7] Eberl G Colonna M Di Santo JP McKenzie AN. Innate lymphoid cells. innate lymphoid cells: a new paradigm in immunology. Science (2015) 348(6237):aaa6566. 25999512 10.1126/science.aaa6566 PMC5658207

[8] Spits H Artis D Colonna M Diefenbach A Di Santo JP Eberl G. Innate lymphoid cells–a proposal for uniform nomenclature. Nat Rev Immunol (2013) 13(2):145–9. doi: 10.1038/nri3365 23348417

[9] Langers I Renoux VM Thiry M Delvenne P Jacobs N. Natural killer cells: role in local tumor growth and metastasis. Biologics (2012) 6:73–82. 22532775 10.2147/BTT.S23976 PMC3333822

[10] Vivier E Tomasello E Baratin M Walzer T Ugolini S. Functions of natural killer cells. Nat Immunol (2008) 9(5):503–10. doi: 10.1038/ni1582 18425107

[11] Kiessling R Klein E Pross H Wigzell H. "Natural" killer cells in the mouse. II. cytotoxic cells with specificity for mouse moloney leukemia cells. characteristics of the killer cell. Eur J Immunol (1975) 5(2):117–21. 10.1002/eji.1830050209 1086218

[12] Kiessling R Klein E Wigzell H. "Natural" killer cells in the mouse. i. cytotoxic cells with specificity for mouse moloney leukemia cells. specificity and distribution according to genotype. Eur J Immunol (1975) 5(2):112–7. 10.1002/eji.1830050208 1234049

[13] Rosenberg EB McCoy JL Green SS Donnelly FC Siwarski DF Levine PH. Destruction of human lymphoid tissue-culture cell lines by human peripheral lymphocytes in 51Cr-release cellular cytotoxicity assays. J Natl Cancer Inst (1974) 52(2):345–52. doi: 10.1093/jnci/52.2.345 4131425

[14] Cerottini JC Brunner KT. Cell-mediated cytotoxicity, allograft rejection, and tumor immunity. Adv Immunol (1974) 18:67–132. doi: 10.1016/S0065-2776(08)60308-9 4151339

[15] Evans R Alexander P. Mechanism of immunologically specific killing of tumour cells by macrophages. Nature (1972) 236(5343):168–70. doi: 10.1038/236168a0 4553694

[16] Norbury KC Fidler IJ. *In vitro* tumor cell destruction by syngeneic mouse macrophoages: methods for assaying cytotoxicity. J Immunol Methods (1975) 7(1):109–22. doi: 10.1016/0022-1759(75)90136-2 1123549

[17] Vosshenrich CA Di Santo JP. Developmental programming of natural killer and innate lymphoid cells. Curr Opin Immunol (2013) 25(2):130–8. doi: 10.1016/j.coi.2013.02.002 23490162

[18] Mombaerts P Iacomini J Johnson RS Herrup K Tonegawa S Papaioannou VE. RAG-1-deficient mice have no mature b and T lymphocytes. Cell (1992) 68(5):869–77. doi: 10.1016/0092-8674(92)90030-G 1547488

[19] Shinkai Y Rathbun G Lam KP Oltz EM Stewart V Mendelsohn M. RAG-2-deficient mice lack mature lymphocytes owing to inability to initiate V(D)J rearrangement. Cell (1992) 68(5):855–67. doi: 10.1016/0092-8674(92)90029-C 1547487

[20] Barrow AD Edeling MA Trifonov V Luo J Goyal P Bohl B. Natural killer cells control tumor growth by sensing a growth factor. Cell (2018) 172(3):534–48.e19. doi: 10.1016/j.cell.2017.11.037 29275861 PMC6684025

[21] Guma M Budt M Saez A Brckalo T Hengel H Angulo A. Expansion of CD94/NKG2C+ NK cells in response to human cytomegalovirus-infected fibroblasts. Blood (2006) 107(9):3624–31. doi: 10.1182/blood-2005-09-3682 16384928

[22] Schmidt S Tramsen L Hanisch M Latge JP Huenecke S Koehl U. Human natural killer cells exhibit direct activity against aspergillus fumigatus hyphae, but not against resting conidia. J Infect Dis (2011) 203(3):430–5. doi: 10.1093/infdis/jiq062 PMC3071101 21208932

[23] Vankayalapati R Garg A Porgador A Griffith DE Klucar P Safi H. Role of NK cell-activating receptors and their ligands in the lysis of mononuclear phagocytes infected with an intracellular bacterium. J Immunol (2005) 175(7):4611–7. doi: 10.4049/jimmunol.175.7.4611 16177106

[24] Mandal A Viswanathan C. Natural killer cells: in health and disease. Hematol Oncol Stem Cell Ther (2015) 8(2):47–55. doi: 10.1016/j.hemonc.2014.11.006 25571788

[25] Caligiuri MA. Human natural killer cells. Blood (2008) 112(3):461–9. doi: 10.1182/blood-2007-09-077438 PMC2481557 18650461

[26] Carrega P Ferlazzo G. Natural killer cell distribution and trafficking in human tissues. Front Immunol (2012) 3:347. doi: 10.3389/fimmu.2012.00347 23230434 PMC3515878

[27] Cooper MA Fehniger TA Caligiuri MA. The biology of human natural killer-cell subsets. Trends Immunol (2001) 22(11):633–40. doi: 10.1016/S1471-4906(01)02060-9 11698225

[28] Nagler A Lanier LL Cwirla S Phillips JH. Comparative studies of human FcRIII-positive and negative natural killer cells. J Immunol (1989) 143(10):3183–91. doi: 10.4049/jimmunol.143.10.3183 2530273

[29] Cooper MA Fehniger TA Turner SC Chen KS Ghaheri BA Ghayur T. Human natural killer cells: a unique innate immunoregulatory role for the CD56(bright) subset. Blood (2001) 97(10):3146–51. doi: 10.1182/blood.V97.10.3146 11342442

[30] Milush JM Long BR Snyder-Cappione JE Cappione AJ 3rd York VA Ndhlovu LC. Functionally distinct subsets of human NK cells and monocyte/DC-like cells identified by coexpression of CD56, CD7, and CD4. Blood (2009) 114(23):4823–31. doi: 10.1182/blood-2009-04-216374 PMC2786291 19805616

[31] Horowitz A Strauss-Albee DM Leipold M Kubo J Nemat-Gorgani N Dogan OC. Genetic and environmental determinants of human NK cell diversity revealed by mass cytometry. Sci Transl Med (2013) 5(208):208ra145. doi: 10.1126/scitranslmed.3006702 PMC3918221 24154599

[32] Karre K Ljunggren HG Piontek G Kiessling R. Selective rejection of h-2-deficient lymphoma variants suggests alternative immune defence strategy. Nature (1986) 319(6055):675–8. doi: 10.1038/319675a0 3951539

[33] Moretta A Bottino C Vitale M Pende D Biassoni R Mingari MC. Receptors for HLA class-I molecules in human natural killer cells. Annu Rev Immunol (1996) 14:619–48. doi: 10.1146/annurev.immunol.14.1.619 8717527

[34] Moretta A Vitale M Bottino C Orengo AM Morelli L Augugliaro R. P58 molecules as putative receptors for major histocompatibility complex (MHC) class I molecules in human natural killer (NK) cells. anti-p58 antibodies reconstitute lysis of MHC class I-protected cells in NK clones displaying different specificities. J Exp Med (1993) 178(2):597–604. 8340759 10.1084/jem.178.2.597 PMC2191136

[35] Pende D Parolini S Pessino A Sivori S Augugliaro R Morelli L. Identification and molecular characterization of NKp30, a novel triggering receptor involved in natural cytotoxicity mediated by human natural killer cells. J Exp Med (1999) 190(10):1505–16. doi: 10.1084/jem.190.10.1505 PMC2195691 10562324

[36] Pessino A Sivori S Bottino C Malaspina A Morelli L Moretta L. Molecular cloning of NKp46: a novel member of the immunoglobulin superfamily involved in triggering of natural cytotoxicity. J Exp Med (1998) 188(5):953–60. doi: 10.1084/jem.188.5.953 PMC3207313 9730896

[37] Sivori S Vitale M Morelli L Sanseverino L Augugliaro R Bottino C. p46, a novel natural killer cell-specific surface molecule that mediates cell activation. J Exp Med (1997) 186(7):1129–36. doi: 10.1084/jem.186.7.1129 PMC2211712 9314561

[38] Vitale M Bottino C Sivori S Sanseverino L Castriconi R Marcenaro E. NKp44, a novel triggering surface molecule specifically expressed by activated natural killer cells, is involved in non-major histocompatibility complex-restricted tumor cell lysis. J Exp Med (1998) 187(12):2065–72. doi: 10.1084/jem.187.12.2065 PMC2212362 9625766

[39] Glasner A Ghadially H Gur C Stanietsky N Tsukerman P Enk J. Recognition and prevention of tumor metastasis by the NK receptor NKp46/NCR1. J Immunol (2012) 188(6):2509–15. doi: 10.4049/jimmunol.1102461 22308311

[40] Halfteck GG Elboim M Gur C Achdout H Ghadially H Mandelboim O. Enhanced *in vivo* growth of lymphoma tumors in the absence of the NK-activating receptor NKp46/NCR1. J Immunol (2009) 182(4):2221–30. doi: 10.4049/jimmunol.0801878 19201876

[41] Lakshmikanth T Burke S Ali TH Kimpfler S Ursini F Ruggeri L. NCRs and DNAM-1 mediate NK cell recognition and lysis of human and mouse melanoma cell lines *in vitro* and *in vivo* . J Clin Invest (2009) 119(5):1251–63. doi: 10.1172/JCI36022 PMC2673866 19349689

[42] Elhaik-Goldman S Kafka D Yossef R Hadad U Elkabets M Vallon-Eberhard A. The natural cytotoxicity receptor 1 contribution to early clearance of streptococcus pneumoniae and to natural killer-macrophage cross talk. PLoS One (2011) 6(8):e23472. doi: 10.1371/journal.pone.0023472 21887255 PMC3161738

[43] Gazit R Gruda R Elboim M Arnon TI Katz G Achdout H. Lethal influenza infection in the absence of the natural killer cell receptor gene Ncr1. Nat Immunol (2006) 7(5):517–23. doi: 10.1038/ni1322 16565719

[44] Carretero M Palmieri G Llano M Tullio V Santoni A Geraghty DE. Specific engagement of the CD94/NKG2-a killer inhibitory receptor by the HLA-e class ib molecule induces SHP-1 phosphatase recruitment to tyrosine-phosphorylated NKG2-a: evidence for receptor function in heterologous transfectants. Eur J Immunol (1998) 28(4):1280–91. doi: 10.1002/(SICI)1521-4141(199804)28:04<1280::AID-IMMU1280>3.0.CO;2-O 9565368

[45] Fan QR Mosyak L Winter CC Wagtmann N Long EO Wiley DC. Structure of the inhibitory receptor for human natural killer cells resembles haematopoietic receptors. Nature (1997) 389(6646):96–100. doi: 10.1038/38028 9288975

[46] Wagtmann N Biassoni R Cantoni C Verdiani S Malnati MS Vitale M. Molecular clones of the p58 NK cell receptor reveal immunoglobulin-related molecules with diversity in both the extra- and intracellular domains. Immunity (1995) 2(5):439–49. doi: 10.1016/1074-7613(95)90025-X 7749980

[47] Wagtmann N Rajagopalan S Winter CC Peruzzi M Long EO. Killer cell inhibitory receptors specific for HLA-c and HLA-b identified by direct binding and by functional transfer. Immunity (1995) 3(6):801–9. doi: 10.1016/1074-7613(95)90069-1 8777725

[48] Torkar M Norgate Z Colonna M Trowsdale J Wilson MJ. Isotypic variation of novel immunoglobulin-like transcript/killer cell inhibitory receptor loci in the leukocyte receptor complex. Eur J Immunol (1998) 28(12):3959–67. doi: 10.1002/(SICI)1521-4141(199812)28:12<3959::AID-IMMU3959>3.0.CO;2-2 9862332

[49] Moretta A Sivori S Vitale M Pende D Morelli L Augugliaro R. Existence of both inhibitory (p58) and activatory (p50) receptors for HLA-c molecules in human natural killer cells. J Exp Med (1995) 182(3):875–84. doi: 10.1084/jem.182.3.875 PMC2192157 7650491

[50] Colonna M Samaridis J. Cloning of immunoglobulin-superfamily members associated with HLA-c and HLA-b recognition by human natural killer cells. Science (1995) 268(5209):405–8. doi: 10.1126/science.7716543 7716543

[51] Bottino C Sivori S Vitale M Cantoni C Falco M Pende D. A novel surface molecule homologous to the p58/p50 family of receptors is selectively expressed on a subset of human natural killer cells and induces both triggering of cell functions and proliferation. Eur J Immunol (1996) 26(8):1816–24. doi: 10.1002/eji.1830260823 8765026

[52] Welte S Kuttruff S Waldhauer I Steinle A. Mutual activation of natural killer cells and monocytes mediated by NKp80-AICL interaction. Nat Immunol (2006) 7(12):1334–42. doi: 10.1038/ni1402 17057721

[53] Le Drean E Vely F Olcese L Cambiaggi A Guia S Krystal G. Inhibition of antigen-induced T cell response and antibody-induced NK cell cytotoxicity by NKG2A: association of NKG2A with SHP-1 and SHP-2 protein-tyrosine phosphatases. Eur J Immunol (1998) 28(1):264–76. doi: 10.1002/(SICI)1521-4141(199801)28:01<264::AID-IMMU264>3.0.CO;2-O 9485206

[54] Lanier LL Corliss B Wu J Phillips JH. Association of DAP12 with activating CD94/NKG2C NK cell receptors. Immunity (1998) 8(6):693–701. doi: 10.1016/S1074-7613(00)80574-9 9655483

[55] Bauer S Groh V Wu J Steinle A Phillips JH Lanier LL. Activation of NK cells and T cells by NKG2D, a receptor for stress-inducible MICA. Science (1999) 285(5428):727–9. doi: 10.1126/science.285.5428.727 10426993

[56] Glienke J Sobanov Y Brostjan C Steffens C Nguyen C Lehrach H. The genomic organization of NKG2C, e, f, and d receptor genes in the human natural killer gene complex. Immunogenetics (1998) 48(3):163–73. doi: 10.1007/s002510050420 9683661

[57] Bellora F Castriconi R Dondero A Reggiardo G Moretta L Mantovani A. The interaction of human natural killer cells with either unpolarized or polarized macrophages results in different functional outcomes. Proc Natl Acad Sci USA (2010) 107(50):21659–64. doi: 10.1073/pnas.1007654108 PMC3003022 21118979

[58] Degli-Esposti MA Smyth MJ. Close encounters of different kinds: dendritic cells and NK cells take centre stage. Nat Rev Immunol (2005) 5(2):112–24. doi: 10.1038/nri1549 15688039

[59] Shanker A Buferne M Schmitt-Verhulst AM. Cooperative action of CD8 T lymphocytes and natural killer cells controls tumour growth under conditions of restricted T-cell receptor diversity. Immunology (2010) 129(1):41–54. doi: 10.1111/j.1365-2567.2009.03150.x 20050329 PMC2807485

[60] Ferlazzo G Tsang ML Moretta L Melioli G Steinman RM Munz C. Human dendritic cells activate resting natural killer (NK) cells and are recognized *via* the NKp30 receptor by activated NK cells. J Exp Med (2002) 195(3):343–51. doi: 10.1084/jem.20011149 PMC2193591 11828009

[61] Gerosa F Baldani-Guerra B Nisii C Marchesini V Carra G Trinchieri G. Reciprocal activating interaction between natural killer cells and dendritic cells. J Exp Med (2002) 195(3):327–33. doi: 10.1084/jem.20010938 PMC2193595 11828007

[62] O'Leary JG Goodarzi M Drayton DL von Andrian UH. T Cell- and b cell-independent adaptive immunity mediated by natural killer cells. Nat Immunol (2006) 7(5):507–16. doi: 10.1038/ni1332 16617337

[63] Paust S Gill HS Wang BZ Flynn MP Moseman EA Senman B. Critical role for the chemokine receptor CXCR6 in NK cell-mediated antigen-specific memory of haptens and viruses. Nat Immunol (2010) 11(12):1127–35. doi: 10.1038/ni.1953 PMC2982944 20972432

[64] Cooper MA Elliott JM Keyel PA Yang L Carrero JA Yokoyama WM. Cytokine-induced memory-like natural killer cells. Proc Natl Acad Sci USA (2009) 106(6):1915–9. doi: 10.1073/pnas.0813192106 PMC2644138 19181844

[65] Bancroft GJ Shellam GR Chalmer JE. Genetic influences on the augmentation of natural killer (NK) cells during murine cytomegalovirus infection: correlation with patterns of resistance. J Immunol (1981) 126(3):988–94. doi: 10.4049/jimmunol.126.3.988 6257788

[66] Biron CA Byron KS Sullivan JL. Severe herpesvirus infections in an adolescent without natural killer cells. N Engl J Med (1989) 320(26):1731–5. doi: 10.1056/NEJM198906293202605 2543925

[67] Bukowski JF Woda BA Habu S Okumura K Welsh RM. Natural killer cell depletion enhances virus synthesis and virus-induced hepatitis *in vivo* . J Immunol (1983) 131(3):1531–8. doi: 10.4049/jimmunol.131.3.1531 6309965

[68] Bukowski JF Woda BA Welsh RM. Pathogenesis of murine cytomegalovirus infection in natural killer cell-depleted mice. J Virol (1984) 52(1):119–28. doi: 10.1128/jvi.52.1.119-128.1984 PMC254497 6207307

[69] Shellam GR Allan JE Papadimitriou JM Bancroft GJ. Increased susceptibility to cytomegalovirus infection in beige mutant mice. Proc Natl Acad Sci USA (1981) 78(8):5104–8. doi: 10.1073/pnas.78.8.5104 PMC320341 6272291

[70] Smith HR Heusel JW Mehta IK Kim S Dorner BG Naidenko OV. Recognition of a virus-encoded ligand by a natural killer cell activation receptor. Proc Natl Acad Sci USA (2002) 99(13):8826–31. doi: 10.1073/pnas.092258599 PMC124383 12060703

[71] Beaulieu AM Zawislak CL Nakayama T Sun JC. The transcription factor Zbtb32 controls the proliferative burst of virus-specific natural killer cells responding to infection. Nat Immunol (2014) 15(6):546–53. doi: 10.1038/ni.2876 PMC4404304 24747678

[72] Karo JM Schatz DG Sun JC. The RAG recombinase dictates functional heterogeneity and cellular fitness in natural killer cells. Cell (2014) 159(1):94–107. doi: 10.1016/j.cell.2014.08.026 25259923 PMC4371485

[73] Madera S Sun JC. Cutting edge: stage-specific requirement of IL-18 for antiviral NK cell expansion. J Immunol (2015) 194(4):1408–12. doi: 10.4049/jimmunol.1402001 PMC4323636 25589075

[74] Min-Oo G Bezman NA Madera S Sun JC Lanier LL. Proapoptotic bim regulates antigen-specific NK cell contraction and the generation of the memory NK cell pool after cytomegalovirus infection. J Exp Med (2014) 211(7):1289–96. doi: 10.1084/jem.20132459 PMC4076589 24958849

[75] O'Sullivan TE Johnson LR Kang HH Sun JC. BNIP3- and BNIP3L-mediated mitophagy promotes the generation of natural killer cell memory. Immunity (2015) 43(2):331–42. doi: 10.1016/j.immuni.2015.07.012 PMC5737626 26253785

[76] Sun JC Madera S Bezman NA Beilke JN Kaplan MH Lanier LL. Proinflammatory cytokine signaling required for the generation of natural killer cell memory. J Exp Med (2012) 209(5):947–54. doi: 10.1084/jem.20111760 PMC3348098 22493516

[77] Sun JC Beilke JN Lanier LL. Adaptive immune features of natural killer cells. Nature (2009) 457(7229):557–61. doi: 10.1038/nature07665 PMC2674434 19136945

[78] Sun JC Beilke JN Lanier LL. Immune memory redefined: characterizing the longevity of natural killer cells. Immunol Rev (2010) 236:83–94. doi: 10.1111/j.1600-065X.2010.00900.x 20636810 PMC2907527

[79] Hammer Q Ruckert T Borst EM Dunst J Haubner A Durek P. Peptide-specific recognition of human cytomegalovirus strains controls adaptive natural killer cells. Nat Immunol (2018) 19(5):453–63. doi: 10.1038/s41590-018-0082-6 29632329

[80] Guma M Cabrera C Erkizia I Bofill M Clotet B Ruiz L. Human cytomegalovirus infection is associated with increased proportions of NK cells that express the CD94/NKG2C receptor in aviremic HIV-1-positive patients. J Infect Dis (2006) 194(1):38–41. doi: 10.1086/504719 16741880

[81] Bjorkstrom NK Lindgren T Stoltz M Fauriat C Braun M Evander M. Rapid expansion and long-term persistence of elevated NK cell numbers in humans infected with hantavirus. J Exp Med (2011) 208(1):13–21. doi: 10.1084/jem.20100762 21173105 PMC3023129

[82] Brunetta E Fogli M Varchetta S Bozzo L Hudspeth KL Marcenaro E. Chronic HIV-1 viremia reverses NKG2A/NKG2C ratio on natural killer cells in patients with human cytomegalovirus co-infection. AIDS (2010) 24(1):27–34. doi: 10.1097/QAD.0b013e3283328d1f 19910789

[83] Gregson JN Kuri-Cervantes L Mela CM Gazzard BG Bower M Goodier MR. Short communication: NKG2C+ NK cells contribute to increases in CD16+CD56- cells in HIV type 1+ individuals with high plasma viral load. AIDS Res Hum Retroviruses (2013) 29(1):84–8. doi: 10.1089/aid.2011.0397 22920222

[84] Oliviero B Varchetta S Paudice E Michelone G Zaramella M Mavilio D. Natural killer cell functional dichotomy in chronic hepatitis b and chronic hepatitis c virus infections. Gastroenterology (2009) 137(3):1151–60, 60.e1-7. doi: 10.1053/j.gastro.2009.05.047 19470388

[85] Petitdemange C Becquart P Wauquier N Beziat V Debre P Leroy EM. Unconventional repertoire profile is imprinted during acute chikungunya infection for natural killer cells polarization toward cytotoxicity. PLoS Pathog (2011) 7(9):e1002268. doi: 10.1371/journal.ppat.1002268 21966274 PMC3178577

[86] Saghafian-Hedengren S Sohlberg E Theorell J Carvalho-Queiroz C Nagy N Persson JO. Epstein-Barr Virus coinfection in children boosts cytomegalovirus-induced differentiation of natural killer cells. J Virol (2013) 87(24):13446–55. doi: 10.1128/JVI.02382-13 PMC3838261 24089567

[87] Guma M Angulo A Vilches C Gomez-Lozano N Malats N Lopez-Botet M. Imprint of human cytomegalovirus infection on the NK cell receptor repertoire. Blood (2004) 104(12):3664–71. doi: 10.1182/blood-2004-05-2058 15304389

[88] Miletic A Krmpotic A Jonjic S. The evolutionary arms race between NK cells and viruses: who gets the short end of the stick? Eur J Immunol (2013) 43(4):867–77. doi: 10.1002/eji.201243101 23440773

[89] Gabor F Jahn G Sedmak DD Sinzger C. *In vivo* downregulation of MHC class I molecules by HCMV occurs during all phases of viral replication but is not always complete. Front Cell Infect Microbiol (2020) 10:283. doi: 10.3389/fcimb.2020.00283 32596168 PMC7304332

[90] Hengel H Koopmann JO Flohr T Muranyi W Goulmy E Hammerling GJ. A viral ER-resident glycoprotein inactivates the MHC-encoded peptide transporter. Immunity (1997) 6(5):623–32. doi: 10.1016/S1074-7613(00)80350-7 9175840

[91] Ahn K Angulo A Ghazal P Peterson PA Yang Y Fruh K. Human cytomegalovirus inhibits antigen presentation by a sequential multistep process. Proc Natl Acad Sci USA (1996) 93(20):10990–5. doi: 10.1073/pnas.93.20.10990 PMC38271 8855296

[92] van der Wal FJ Kikkert M Wiertz E. The HCMV gene products US2 and US11 target MHC class I molecules for degradation in the cytosol. Curr Top Microbiol Immunol (2002) 269:37–55. doi: 10.1007/978-3-642-59421-2_3 12224515

[93] Beck S Barrell BG. Human cytomegalovirus encodes a glycoprotein homologous to MHC class-I antigens. Nature (1988) 331(6153):269–72. doi: 10.1038/331269a0 2827039

[94] Chapman TL Heikeman AP Bjorkman PJ. The inhibitory receptor LIR-1 uses a common binding interaction to recognize class I MHC molecules and the viral homolog UL18. Immunity (1999) 11(5):603–13. doi: 10.1016/S1074-7613(00)80135-1 10591185

[95] Farrell HE Vally H Lynch DM Fleming P Shellam GR Scalzo AA. Inhibition of natural killer cells by a cytomegalovirus MHC class I homologue *in vivo* . Nature (1997) 386(6624):510–4. doi: 10.1038/386510a0 9087412

[96] Wang EC McSharry B Retiere C Tomasec P Williams S Borysiewicz LK. UL40-mediated NK evasion during productive infection with human cytomegalovirus. Proc Natl Acad Sci USA (2002) 99(11):7570–5. doi: 10.1073/pnas.112680099 PMC124287 12032324

[97] Kotenko SV Saccani S Izotova LS Mirochnitchenko OV Pestka S. Human cytomegalovirus harbors its own unique IL-10 homolog (cmvIL-10). Proc Natl Acad Sci USA (2000) 97(4):1695–700. doi: 10.1073/pnas.97.4.1695 PMC26498 10677520

[98] Chang WL Baumgarth N Yu D Barry PA. Human cytomegalovirus-encoded interleukin-10 homolog inhibits maturation of dendritic cells and alters their functionality. J Virol (2004) 78(16):8720–31. doi: 10.1128/JVI.78.16.8720-8731.2004 PMC479089 15280480

[99] Holder KA Grant MD. Human cytomegalovirus IL-10 augments NK cell cytotoxicity. J Leukoc Biol (2019) 106(2):447–54. doi: 10.1002/JLB.2AB0418-158RR 30964577

[100] Beziat V Liu LL Malmberg JA Ivarsson MA Sohlberg E Bjorklund AT. NK cell responses to cytomegalovirus infection lead to stable imprints in the human KIR repertoire and involve activating KIRs. Blood (2013) 121(14):2678–88. doi: 10.1182/blood-2012-10-459545 PMC3617633 23325834

[101] Green ML Leisenring WM Xie H Walter RB Mielcarek M Sandmaier BM. CMV reactivation after allogeneic HCT and relapse risk: evidence for early protection in acute myeloid leukemia. Blood (2013) 122(7):1316–24. doi: 10.1182/blood-2013-02-487074 PMC3744995 23744585

[102] Ito S Pophali P Co W Koklanaris EK Superata J Fahle GA. CMV reactivation is associated with a lower incidence of relapse after allo-SCT for CML. Bone Marrow Transpl (2013) 48(10):1313–6. doi: 10.1038/bmt.2013.49 PMC5274543 23562969

[103] Basilio-Queiros D Venturini L Luther-Wolf S Dammann E Ganser A Stadler M. Adaptive NK cells undergo a dynamic modulation in response to human cytomegalovirus and recruit T cells in *in vitro* migration assays. Bone Marrow Transpl (2022) 57(5):712–20. doi: 10.1038/s41409-022-01603-y PMC9090630 35177828

[104] Schlums H Cichocki F Tesi B Theorell J Beziat V Holmes TD. Cytomegalovirus infection drives adaptive epigenetic diversification of NK cells with altered signaling and effector function. Immunity (2015) 42(3):443–56. doi: 10.1016/j.immuni.2015.02.008 PMC4612277 25786176

[105] Lee J Zhang T Hwang I Kim A Nitschke L Kim M. Epigenetic modification and antibody-dependent expansion of memory-like NK cells in human cytomegalovirus-infected individuals. Immunity (2015) 42(3):431–42. doi: 10.1016/j.immuni.2015.02.013 PMC4537797 25786175

[106] Dickinson RE Milne P Jardine L Zandi S Swierczek SI McGovern N. The evolution of cellular deficiency in GATA2 mutation. Blood (2014) 123(6):863–74. doi: 10.1182/blood-2013-07-517151 PMC3916878 24345756

[107] Spinner MA Sanchez LA Hsu AP Shaw PA Zerbe CS Calvo KR. GATA2 deficiency: a protean disorder of hematopoiesis, lymphatics, and immunity. Blood (2014) 123(6):809–21. doi: 10.1182/blood-2013-07-515528 PMC3916876 24227816

[108] Schlums H Jung M Han H Theorell J Bigley V Chiang SC. Adaptive NK cells can persist in patients with GATA2 mutation depleted of stem and progenitor cells. Blood (2017) 129(14):1927–39. doi: 10.1182/blood-2016-08-734236 PMC5383869 28209719

[109] Brodsky RA. Paroxysmal nocturnal hemoglobinuria. Blood (2014) 124(18):2804–11. doi: 10.1182/blood-2014-02-522128 PMC4215311 25237200

[110] Corat MA Schlums H Wu C Theorell J Espinoza DA Sellers SE. Acquired somatic mutations in PNH reveal long-term maintenance of adaptive NK cells independent of HSPCs. Blood (2017) 129(14):1940–6. doi: 10.1182/blood-2016-08-734285 PMC5383870 27903532

[111] Mody CH Ogbomo H Xiang RF Kyei SK Feehan D Islam A. Microbial killing by NK cells. J Leukoc Biol (2019) 105(6):1285–96. doi: 10.1002/JLB.MR0718-298R 30821868

[112] Erkeller-Yuksel FM Lydyard PM Isenberg DA. Lack of NK cells in lupus patients with renal involvement. Lupus (1997) 6(9):708–12. doi: 10.1177/096120339700600905 9412985

[113] Erkeller-Yusel F Hulstaart F Hannet I Isenberg D Lydyard P. Lymphocyte subsets in a large cohort of patients with systemic lupus erythematosus. Lupus (1993) 2(4):227–31. doi: 10.1177/0961203393002001081 8268970

[114] Schepis D Gunnarsson I Eloranta ML Lampa J Jacobson SH Karre K. Increased proportion of CD56bright natural killer cells in active and inactive systemic lupus erythematosus. Immunology (2009) 126(1):140–6. doi: 10.1111/j.1365-2567.2008.02887.x PMC2632704 18564343

[115] Dalbeth N Callan MF. A subset of natural killer cells is greatly expanded within inflamed joints. Arthritis Rheumatol (2002) 46(7):1763–72. doi: 10.1002/art.10410 12124859

[116] Gur C Porgador A Elboim M Gazit R Mizrahi S Stern-Ginossar N. The activating receptor NKp46 is essential for the development of type 1 diabetes. Nat Immunol (2010) 11(2):121–8. doi: 10.1038/ni.1834 20023661

[117] Brandau S Riemensberger J Jacobsen M Kemp D Zhao W Zhao X. NK cells are essential for effective BCG immunotherapy. Int J Cancer (2001) 92(5):697–702. doi: 10.1002/1097-0215(20010601)92:5<697::AID-IJC1245>3.0.CO;2-Z 11340575

[118] Rydyznski CE Waggoner SN. Boosting vaccine efficacy the natural (killer) way. Trends Immunol (2015) 36(9):536–46. doi: 10.1016/j.it.2015.07.004 PMC4567442 26272882

